# Editorial: The social brain: new insights from social, clinical, and biological psychology

**DOI:** 10.3389/fpsyg.2025.1552456

**Published:** 2025-01-29

**Authors:** Carmelo M. Vicario, Chiara Lucifora, Giuseppe Craparo, Paola Magnano, Gabriella Martino

**Affiliations:** ^1^Dipartimento di Scienze Cognitive, Psicologiche, Pedagogiche e Degli Studi Culturali, Università di Messina, Messina, Italy; ^2^Dipartimento di Filosofia e Comunicazione, Università di Bologna, Bologna, Italy; ^3^Faculty of Human and Social Sciences, Kore University of Enna, Cittadella Universitaria, Enna, Italy; ^4^Department of Clinical and Experimental Medicine, University of Messina, Messina, Italy

**Keywords:** social brain, clinical psychology, biological psychology, social psychology, neurosciece

The concept of the “social brain” encapsulates the intricate interplay between neural processes and social behaviors, providing a framework for understanding how we navigate our social world. This is suggested by the literature on brain disorders (Vicario and Lucifora, 2021; D'Amico et al., 2024), as well as research on healthy individuals that highlights the role of personality traits (Rinella et al., 2019; Massimino et al., 2019) and coping strategies (Massimino et al., 2024; Rinella et al., 2017).

The recent collection of articles published in *Frontiers in Psychology* under the Research Topic “*The Social Brain: New Insights from Social, Clinical, and Biological Psychology*” presents a diverse range of studies that deepen our understanding of this multifaceted domain.

Zhang, Cai, et al. examined the impact of moral judgment on bystanders' interpersonal trust, identifying trustworthiness as a crucial mediating factor. This research underscores how moral evaluations can significantly influence social relationships and community interactions. Notably, Vicario et al. (2018) demonstrated that hunger and satiety can affect the judgment of ethical violations, suggesting that physiological states can shape moral reasoning and social cognition. Xu et al. investigated the relationship between cognitive reappraisal, empathy, and prosocial behavior in adolescents. Their findings highlight the importance of emotional regulation strategies in fostering empathetic responses, which are essential for constructive social interactions. This is in line with the earlier work by Vicario et al. (2023) providing evidence of altered fear extinction learning in individuals with high vaccine hesitancy during the COVID-19 pandemic, which underscores the significant role that anxiety and emotional regulation play in public health decisions and social behavior. Complementing this, Troncoso et al. focused on the dynamics of empathy by examining sensorimotor and physiological responses during synchronous experiences of suffering, contributing to our understanding of embodied empathy in social contexts.

Zhang, Deng, et al. conducted a systematic review analyzing trustworthiness studies using the Web of Science database, providing critical insights into how trust and social perception are studied across various disciplines. A further systematic review/meta-analysis was conducted by Dai et al. investigating neural mechanisms of different types of envy.

Guingrich and Graziano explored the implications of attributing consciousness to artificial intelligence, revealing how human-AI interactions can influence subsequent human-to-human interactions. This study offers a novel perspective on the evolving landscape of social cognition in the digital age.

Li et al. examined cognitive control mechanisms involved in honesty and dishonesty across various conflict scenarios, shedding light on the cognitive processes that govern ethical decision-making. In a related study, Lucifora et al. (2021) demonstrated how self-control predicts moral decision-making, showing that individuals with higher self-control exhibit greater ethical considerations in their choices. This body of work, along with Myznikov et al., which investigated the relationship between dark triad personality traits and structural brain changes, indicates a neurobiological basis for personality in shaping social behavior and ethical judgments.

In conclusion, Armas-Vargas et al. focused on the psychometric properties of the CEMA-A questionnaire, assessing motives for lying, which is crucial for addressing ethical behavior and trust in social interactions. Lastly, Zhang, Li, et al. examined how group membership influences adolescents' third-party punishment behaviors, emphasizing the importance of group identity in moral judgment and social dynamics.

The collection of articles featured in this Research Topic serves as a starting point to the strides being made in our understanding of the social brain. By elucidating the interplay of biological, social, and clinical factors, this collection of studies provides a comprehensive framework that advances scientific knowledge and translates into practical strategies for improving social cognition and connectedness in diverse populations. As we continue to explore the intricate matrices of the social brain, the insights gleaned from this body of work are essential for shaping the future of psychological research and practice.
